# Deep Learning-Based Adaptive Compression and Anomaly Detection for Smart B5G Use Cases Operation

**DOI:** 10.3390/s23021043

**Published:** 2023-01-16

**Authors:** Ahmad El Sayed, Marc Ruiz, Hassan Harb, Luis Velasco

**Affiliations:** 1 Advanced Broadband Communications Center (CCABA), Universitat Politècnica de Catalunya (UPC), 08034 Barcelona, Spain; 2 College of Engineering and Technology, American University of the Middle East, Egaila 54200, Kuwait

**Keywords:** B5G use cases, digital twins, data compression, anomaly detection, autoencoders

## Abstract

The evolution towards next-generation Beyond 5G (B5G) networks will require not only innovation in transport technologies but also the adoption of smarter, more efficient operations of the use cases that are foreseen to be the high consumers of network resources in the next decades. Among different B5G use cases, the Digital Twin (DT) has been identified as a key high bandwidth-demanding use case. The creation and operation of a DT require the continuous collection of an enormous and widely distributed amount of sensor telemetry data which can overwhelm the transport layer. Therefore, the reduction in such transported telemetry data is an essential objective of smart use case operation. Moreover, deep telemetry data analysis, i.e., anomaly detection, can be executed in a hierarchical way to reduce the processing needed to perform such analysis in a centralized way. In this paper, we propose a smart management system consisting of a hierarchical architecture for telemetry sensor data analysis using deep autoencoders (AEs). The system contains AE-based methods for the adaptive compression of telemetry time series data using pools of AEs (called AAC), as well as for anomaly detection in single (called SS-AD) and multiple (called MS-AGD) sensor streams. Numerical results using experimental telemetry data show compression ratios of up to 64% with reconstruction errors of less than 1%, clearly improving upon the benchmark state-of-the-art methods. In addition, fast and accurate anomaly detection is demonstrated for both single and multiple-sensor scenarios. Finally, a great reduction in transport network capacity resources of 50% and more is obtained by smart use case operation for distributed DT scenarios.

## 1. Introduction

Time series data are one of the most predominantly generated in modern information systems [1], with sensors being responsible for a larger portion of such data production. Among several applications requiring exhaustive and extensive sensor data collection, the creation and synchronization of Digital Twins (DT) have been attracting recent and large interest from both academic and industrial sectors [2]. The data generated from this type of application needs to be collected and processed at a high resolution, which entails high monitoring/telemetry frequencies, i.e., sub-second sampling rates. Several examples of DTs for the modeling and management of smart systems can be found in the literature, e.g., for smart manufacturing [3], drinking water management and distribution systems [4], and optical communications [5].

Typically, DTs require the collection of data from widely distributed sensor network systems and their transport to a centralized place, e.g., the cloud, where it is processed. In fact, the DT is a well-known use case of an emerging service that is pushing the evolution of current transport networks towards the beyond 5G (B5G) era [6]. This type of service requires reliable and high-bitrate connectivity between plenty of physical locations holding heterogeneous sources (wireless and wired sensors) and the premises where the DT is built and managed, which commonly entails connectivity across domains including access, metro, and core networks. Therefore, developing solutions to compress these dense monitoring data before their transport is essential to reduce operational costs including connectivity, storage, and energy consumption [7].

Regarding different compression techniques suitable for time series data, two main categories can be identified: lossless and lossy [8]. On the one hand, lossless compression means that the decompressed data are identical to the compressed data, which tends to produce low compression ratios, e.g., 50% compression. On the other hand, lossy compression techniques are intended to produce a trade-off between the accuracy of the reconstructed data and higher compression ratios, e.g., 90% compression. For the latter category, the use of deep learning (DL) methods has attracted special interest from the research community. Among different DL techniques, autoencoders (AE) represent a promising opportunity in the field of lossy compression. Basically, AEs are a type of deep neural network that has an encoder and a decoder part. The encoder part compresses input data into a number of representations called latent features which have a size much smaller than the input dimension. These representations form the latent space (LS) of the AE. The encoder is usually able to compress the data by discarding non-relevant parts of the data while keeping only the parts that can be effectively used for reconstruction in the decoder part. Another advantage of using AEs is their intrinsic ability for anomaly detection [9]. Note that this allows performing not only compression but also data analysis at the source before transport. Thus, locally distributed data analysis can be performed and used to add more intelligence to the monitoring system, e.g., increasing a nominal monitoring sampling rate when an anomaly is detected [10].

In this paper, we propose a novel method for the lossy compression of time series data using deep AEs along with two methods for anomaly detection that operate on both single and multiple time series. For the compression, instead of compressing the input data using a single AE, a pool of AEs with a different number of latent features is used. Thus, the *Adaptive AE-based Compression* (AAC) method is presented as an autonomous process that is able to choose the best AE in the pool, i.e., the one that reaches a target reconstruction error with the minimum LS size. The variability of the number of latent features means that the size of the compressed data is not fixed, which draws similarities between AE-based compression and conventional compression methods in which the characteristics of the input data play an important role in the compression ratio. It also means that the compression is adaptive to the variations in the data and hence, compression size is indeed a variable that can be analyzed as additional and extended information of collected monitoring data. It is worth mentioning that AEs are trained using data from the specific sensor/s that they operate. However, since this may not be available from the beginning of sensor operation, generic AEs with moderated compression rates trained for heterogeneous sensor data are used until enough data are collected to train the specific AEs.

Regarding the anomaly detection part, the first method, called *Single Sensor Anomaly Detection* (SS-AD), takes advantage of the specificity of the trained AEs to detect when the collected data contains an anomaly, e.g., if the sensor malfunctions or some kind of additional noise is introduced to the data from an external source. The second method, called the *Multiple Sensor Anomalous Group Diagnosis* (MS-AGD), detects anomalies that can affect several sensors in a correlated way, even when they cannot be detected by SS-AD in an independent time series. It does this by comparing data points with a certain degree of reconstruction error values across all the time series involved, making it able to detect subtle correlated anomalies.

The remainder of this paper is as follows: Section 2 describes the latest related work on the subject of time series compression and anomaly detection. Section 3 describes the network architecture and main components. Section 4 describes our contribution in terms of the algorithms for compression and anomaly detection. Section 5 shows the performance evaluation in a simulated environment using an experimental data set, including a comparison to state-of-the-art methods. Eventually, Section 6 draws the main conclusions of this research work.

## 2. Related Work

The widespread nature of time series data and the necessity to reduce its continually growing sizes make the task of time series data compression very important, so plenty of work is found in the literature about this topic. In addition, due to its importance in modern systems, such as sensor networks, IoT, and DT systems, anomaly detection in time series data is discussed fairly. Table 1 summarizes the main contributions in the literature on time series compression and anomaly detection.

### 2.1. Lossy Time Series Data Compression

The authors of [11,12] tackled the problem of lossy compression in time series data using different techniques. In [11], a piecewise regression technique is used in order to compress time series data from the smart grid. The approach depended on three regression algorithms, each specializing in a class of polynomial functions, which were applied incrementally. The final compression factor depended on a user-defined maximum tolerable deviation between the original time series and the reconstructed one. The authors of [13] proposed a method of lossy compression that depends on extrema (minimum and maximum) extracted from the data. Different definitions and different importance levels for extrema were applied in several pass algorithms. The authors in [12] performed an evaluation of five data compression algorithms and five change detection algorithms on several datasets. Their approach focused on finding out how these different techniques perform under different datasets with different characteristics, and how best to choose the parameters under which these algorithms will work properly.

Regarding time series compression using AEs, the authors in [14] developed an algorithm called LFZip (*Lossy Floating-point Zip*) which compresses time series by using an encoder and a decoder that is based on the prediction-quantization-entropy coder framework, with works under the mean absolute error metric that have a maximum allowable error that is defined by the user. Another variant of the AE, the Convolutional AE, was used by the authors in [15] to compress and decompress electroencephalogram signals in order to reduce data size, thus conserving the energy of the edge devices reading and transmitting these signals. Another medical application used AEs to compress data collected from wearable IoT data that have a limited energy source [16], using three parameters: compression ratio, reconstruction error, and energy consumption, to optimize the learning process. Furthermore, for IoT applications with limited processing memory, the authors in [17] developed a low memory, low latency algorithm for time series compression that allows decompressing later at speeds up to 3 GB/s by using a high-speed forecasting algorithm. A Recurrent Neural Network (RNN)-based AE was used in [18], combined with data segmentation and aggregation into segments of variable length but with a similar total variation. Similarly, RNN AEs were used in [19] to partially reconstruct multi-dimensional time series data effectively, allowing insight into the operating state of some of the sensors in the system without the need for full reconstruction.

### 2.2. Anomaly Detection in Time Series

A remarkable list of use cases and algorithms for anomaly detection in time series can be found in the literature [20,21]. In [20], the authors used deep AEs trained with raw time series data from flight sensors collected under nominal operating conditions and examined the reconstruction error to detect faults with up to 97% accuracy and identify two types of faults with no false positives. Similarly, deep AEs inspired by the robust principal component analysis were developed in [22] to detect outliers and perform de-noising even without access to clean data. The method proposed in [23], called GGM-VAE, uses a Gated Recurrent Unit (GRU) and is used to discover the correlation in multi-dimensional time series data. Another approach that tackles anomaly detection in multivariate time series is the method described in [24]. It describes the usage of 1D convolutional neural networks, where the convolutions are performed over the inputs across the temporal axis of the data, to detect anomalies in sewer processing monitoring data by checking if the reconstruction error in the decoding stage is above a certain value.

In addition, in the scope of anomaly detection, the authors in [25] used a technique they called smoothness inducing sequential variational AEs (SISVAE), which is based on Variational AEs (VAE) but has a backbone in RNNs. Their method uses the mean and variance of each sample as parameters, which means the compression process is not rigid and is flexible to the variations in the data. Moreover, to compensate for the susceptibility to anomalies that this approach generates, a smoothness-inducing prior over possible estimations is used, thus penalizing non-smooth estimations. The authors in [26] used the Echo-State Network, which is a method used to train RNN where only parameters for output are learned in order to train VAEs to detect anomalies in a multivariate time series, making use of the temporal dependence in the data. A hybrid approach for anomaly detection was used in [27], where Long-Short Term Memory (LSTM)-based AEs trained on normal samples were used to extract features from both normal samples and ones containing anomalies where an SVM classifier is used for detection purposes. A squeezed Convolutional VAE (SCVAE) was modeled to detect anomalies in edge devices of IoT as described in [28], and the reconstruction probability, which is a probabilistic measure that takes into account the variability of the distribution of variables, was used to tune VAEs to detect anomalies in [29]. Finally, the authors in [21] conducted a survey of the anomaly detection methods for time series across a variety of domains and concluded that the main challenges remain the real-time processing, online adaptive learning, multivariate data, the shortage of labels anomaly data, and the difficulty in obtaining it, and the lack of a generalized approach which works in all cases.

**Table 1:** State-of-the-art summary (sorted by year).

Ref	Year	Compression	Anomaly Detection	Processing Power	Summary of Methods
[13]	2011	yes	No	High	Extracting Important Minima and Maxima
[11]	2014	yes	No	High	Piecewise Regression
[29]	2015	no	Yes	High	Reconstruction Probability Analysis
[20]	2016	no	Yes	Low	Deep AEs
[26]	2016	no	Yes	Low	Echo State Training; RNN
[12]	2017	yes	No	-	Evaluating Different Time Series Compression Methods
[18]	2017	yes	No	Medium	Adaptive Piecewise Recurrent AE
[22]	2017	no	Yes	Medium	Deep AE; Principle Component Analysis
[15]	2018	yes	No	Medium	Convolutional AEs
[17]	2018	yes	No	Low	Low Memory, Low Latency Forecasting Algorithm
[19]	2018	yes	No	Low	Recurrent AEs; Partial Reconstruction
[23]	2018	no	yes	Low	Gated Recurrent Units
[28]	2018	no	yes	Low	Squeezed Convolutional AE
[14]	2020	yes	no	Medium	Prediction-Quantization-Entropy Encoder
[24]	2020	no	yes	Low	1D Convolutional Neural Network
[21]	2020	no	yes	-	Survey of several techniques
[27]	2020	no	yes	Medium	LSTM AEs; SVM Classifier
[16]	2020	yes	no	Low	AEs
[25]	2021	no	yes	Medium	Smoothness Inducing Sequential Variational AE

### 2.3. Summary and Contributions

As can be seen, to the best of our knowledge, we can conclude that: (i) none of the methods perform both operations (compression + anomaly detection) at the same time; (ii) some of them require a lot of real-time processing power at the level of the agent performing the compression or the anomaly detection; and (iii) some of them are only deployable after extensive training using data from the targeted systems, which may delay the deployment process.

In this work, we propose a hierarchical architecture for telemetry analysis that enables efficient and adaptive compression (by means of the AAC method) and anomaly detection (by means of SS-AD and MS-AGD methods) simultaneously. Hence, our proposed novel system outperforms the methods in the literature in the following ways: (i) performs both compression and anomaly detection at the same time using the same models based on AEs; (ii) requires very little processing power of the agent by using AEs for both compression and anomaly detection tasks; and (iii) enables immediate deployment by using AEs trained with general-purpose data, which allows performing at acceptable levels of compression and reconstruction errors until enough sensor-specific data are collected to train system-specific AEs.

## 3. AE-Based Telemetry Compression and Anomaly Detection

### 3.1. Concept and Architecture

**Figure 1:**
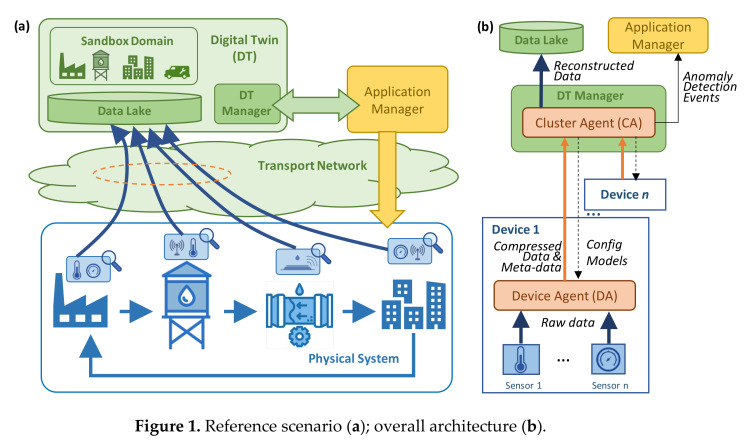
Reference scenario (**a**); overall architecture (**b**).

The reference scenario is sketched in Figure 1a, where a physical system contains a plethora of different sensors that generate heterogeneous telemetry data that need to be gathered and analyzed for several purposes such as smart autonomous operation. Although the example in Figure 1 sketches a water distribution system, the proposed architecture and algorithms are designed to fit with any smart system collecting time series telemetry data such as smart manufacturing and communication networks, just to mention a few. Without loss of generality, let us assume that the sensors generate data periodically, with a fixed time interval (that can be different among sensors). Therefore, every single sensor is a source of one or more time series telemetry data streams. All these data flows need to be transported from their sources to the centralized location where the DT is running. A typical DT architecture consists of three essential components: (i) a *Data Lake*, where the collected, pre-processed, and post-processed data are stored; (ii) the *Sandbox Domain*, containing the different models and algorithms that emulate the different components of the physical system; and (iii) the *Digital Twin Manager* (DTM) that is in charge of several actions including the management of the models in the sandbox domain. Moreover, the DTM interfaces with the Application Manager in charge of both the physical and DT systems. Note that the Application Manager uses the DT to analyze the current and future state of the physical system, which can be achieved by combining the collected data available in the Data Lake and the models and algorithms in the sandbox domain. The result of such analysis can lead to specific actions to be executed in the physical system. Moreover, the Application Manager can configure rules and policies to the DTM, so that the latter can perform tasks such as intelligent data aggregation and anomaly detection in an autonomous way.

Figure 1b provides a deeper insight into the hierarchical architecture needed to run the proposed telemetry data compression and analysis. The first level is at the sensor layer where data are generated periodically. For the sake of simplicity, let us assume that sensors are those physical elements that are able to monitor one specific metric, e.g., temperature, pressure, etc. Then, a number of these sensors are integrated into a monitoring *device*, that provides the support (computing, power) to those sensors, as well as contains the needed transceivers and interfaces (wired or wireless) required to eject the data out of the device. Since the vast majority of multi-purpose monitoring devices are built on top of powerful boards such as Arduino or Raspberry Pi [30,31], a software-based *Device Agent* (DA) is deployed in the device for several purposes, including telemetry data processing and device control and management. Specifically, in the context of our work, we consider that the DA contains the AEs necessary to compress the collected telemetry data and perform anomaly detection. Then, the DA sends the compressed data to the DTM that is hosted in the remote location. Along with the compressed data, three types of metadata are sent: (i) the device/sensors identification data, including location; (ii) the compression method metadata, including aspects such as the AE id that is required to decompress the data, as well as the expected reconstruction error; and (iii) the anomaly detection diagnosis, in case that some anomaly affecting one or multiple sensors is detected.

The second element in the proposed hierarchical architecture is the *Cluster Agent* (CA) which runs as one of the processes in DTM and aggregates the inputs received from a number of devices that form a group (cluster). The meaning of a cluster is open: it can represent any subset of monitoring devices in a physical subsystem. Without loss of generality, we assume that the creation of clusters is part of the design of both the physical system and DT, which is out of the scope of this paper. Each CA is in charge of decompressing the data received from its nested DAs and storing such decompressed data in the Data Lake. Moreover, it is also in charge of training AEs as soon as new relevant data are collected and uploading new models to the DAs in an automatized manner. Finally, it processes the anomaly detection diagnosis reports received from DAs, performs multiple anomaly detection if needed, and notifies the application manager in case of some anomaly event has been detected.

The next subsection presents a detailed architecture of the main building blocks running in DA and CA elements, including the three main processes previously introduced: *Adaptive AE-based Compression* (AAC), *Single Sensor Anomaly Detection* (SS-AD), and *Multiple Sensor Anomalous Group Diagnosis* (MS-AGD).

### 3.2. Main Components

**Figure 2:**
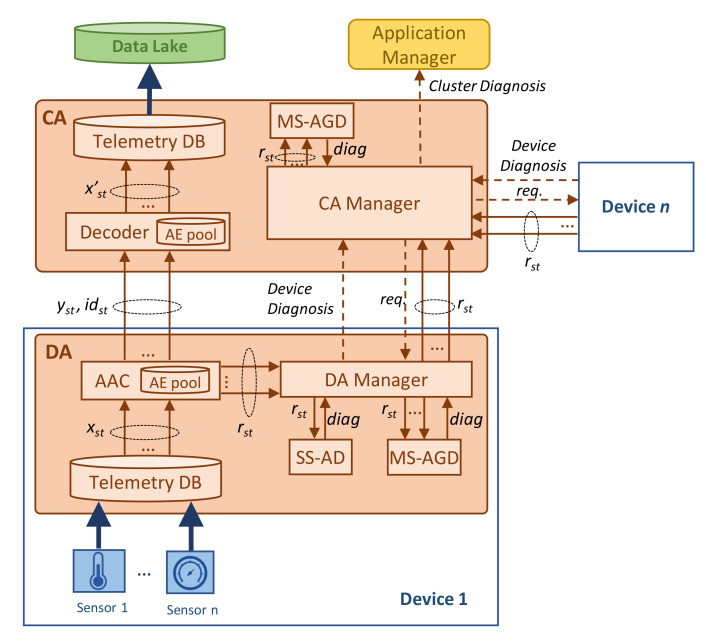
Detailed architecture and key components.

Figure 2 details the architecture previously sketched in Figure 1b, showing the key building blocks and their relationship. The figure focuses on the processes related to telemetry data compression and anomaly detection. For the sake of simplicity, the processes of training and updating AEs are not depicted in the figure. Let us assume that the DA implements a telemetry database (DB) that temporarily stores the data injected by each of the sensors in the device. We can assume that this data collection is accomplished at a very narrow *telemetry interval,* e.g., one measurement per second and device. Then, a larger *monitoring interval,* e.g., every minute, is configured to retrieve data from the telemetry DB and compress them. Thus, let us denote *x_st_* as the telemetry measurements collected during monitoring interval *t* by sensor *s*. These data are then fed to the *compressor* module that is responsible for running the AAC process. By means of the AE pool, adaptive and effective compression is achieved. The compressed telemetry data (denoted as *y_st_*) as well as the identifier of the AE selected by AAC for compression (denoted as *id_st_*) are sent to the CA. Without loss of generality, we assume that CA process the received compressed data immediately upon their reception, calling a simple *de-compressor* process that uses the decoder of the selected AE to reconstruct the original telemetry stream (denoted as *x’_st_*) and inject it into the data lake.

In addition to the compressed telemetry data, the AAC process also computes the reconstruction error vector obtained by the selected AE (denoted as *r_st_*). This error is defined as the difference between the original and reconstructed telemetry measurements. This relevant output is locally processed at the DA for anomaly detection purposes. Specifically, the *DA manager* receives a reconstruction error vector per each sensor and monitoring interval and triggers two different anomaly detection processes. On the one hand, the SS-AD analyzes the individual reconstruction error of each sensor in order to find an anomalous error pattern such as continuous large error. On the other hand, the MS-AGD analyzes the reconstruction errors of the sensors in the device and performs a correlated analysis in order to identify subtle anomalies affecting several sensors at the same time. The diagnosis generated by each of the methods is then processed by the DA manager that generates a device diagnosis report when a remarkable event is detected by one or both methods.

Such device diagnosis report (if generated) is sent to the *CA manager* which can trigger a wider and deeper anomaly analysis. In particular, it can request to the DA of the devices under its control those reconstruction error vectors that have not been sent before. As an illustrative example, let us imagine that an anomaly in a temperature sensor has been detected in device *i*. The CA can then request the reconstruction error of the rest of the temperature sensors of all the devices in the cluster in order to perform a group analysis and detect, e.g., an incipient temperature anomaly in other elements of the system. Note that, to allow this analysis, we consider that DA managers temporarily store reconstruction errors even when they are not detecting any anomaly. Finally, the results of received device diagnosis reports generated by DA managers and the sensor group analysis (if proceeding) generated by the CA manager compose the cluster diagnosis report that is sent to the application manager.

The next section presents detailed algorithms for AAC, SS-AD, and MS-AGD processes.

## 4. Algorithms

### 4.1. Notation

Table 2 provides the main notations that are consistently used in the following algorithms.

### 4.2. AAC

Algorithm 1 details the pseudo-code of the AAC process which runs for each sensor *s* in a device and is executed at every interval *t* a new telemetry stream is available. As introduced in the previous section, it receives the raw telemetry data stream *x_st_* containing a number *w* of measurements, the pool of AEs of the sensor *Ψ_s_*_,_ and the reconstruction error threshold *ε_comp_* to determine whether a given compressed stream *y_st_* produces enough of an accurately reconstructed telemetry stream when decoded. In addition to *y_st_*, the algorithm also returns the identifier of the selected AE in the pool *id_st_*, as well as the reconstruction error vector *r_st_*.

After initializing output variables (line 1 of Algorithm 1), the set of AEs in the pool *Ψ_s_* are sorted in ascendant order of size of LS (line 2). Thus, they are going to be sequentially evaluated in a loop from the highest to lowest compression ratio (line 3). Given an AE *ψ*, the input data are normalized with the min-max values stored as model coefficients (line 4). Then, the normalized input *x* is propagated through the encoder part and the compressed stream *y* is obtained (line 5). At this point, the decoder is used to compute the reconstructed data stream *x’*, which is used to compute the reconstruction error *r* that *ψ* produces (lines 6–7). Note that if the average reconstruction error is below threshold *ε_comp_*, then an accurate compression is found, and the AE pool search is interrupted (lines 8–10). Finally, the resultant output is returned (line 11). Note that this output can be either a compressed telemetry stream if an AE producing an average reconstruction error below *ε_comp_* is found or the original input if no accurate compression can be effectively accomplished.
**Algorithm 1.** AAC method.**INPUT**: *x_s__t_, Ψ_s_, ε_comp_* **OUTPUT**: *y_st_, id_st_, r_st_*1. 2. 3. 4. 5. 6. 7. 8. 9. 10. 11. *y_st_ ← x_s__t_; id_st_ ←* ∅; *r_st_ ←* zeros(*w*) **sort**(*Ψ_s_, |ψ.*latent|, “ascendent”) **for each** *ψ* ∈ *Ψ_s_*:    *x ←* normalize*(x_s__t,_ ψ.*minmax)    *y ← ψ.*encoder.propagate(*x*)    x’ ← ψ.decoder.propagate(*y*)    r← computeReconstructionError(*x*, *x’*)   **if** avg(*r*) *≤ ε_comp_*:        *y_st_ ← y; id_st_ ← ψ.*id; *r_st_ ← r*
      **break**
 **return**
*y_s__t_, id_st_, r_st_*

Recall that we consider that generic AEs with moderated compression rates trained from heterogeneous generic sensor data are used until enough sensor-specific data are collected to train ad-hoc AEs that better compress the data of a given sensor. Without loss of generality, we can assume that this procedure can run periodically as soon as telemetry data from sensors are available. Algorithm 2 details the proposed procedure to train and update the AEs in a pool. Thus, given an AE pool Ψ (that could be initially empty) and a database DB containing telemetry measurements (that can be either generic or sensor-specific), the algorithm trains a set of AEs with LS sizes defined in set Z in order to find new models that improve existing ones.
**Algorithm 2.** AE pool update.**INPUT**: *Ψ_s_, Z, DB* **OUTPUT**: *Ψ_s_*1. 2. 3. 4. 5. 6. 7. 8. 9. 10. 11. 12. 13. 14. 15. 16. *DB_train_*, *DB_test_* ← split(*DB*) **for each** z ∈ *Z*:    *ψ_new_* ← trainAE(*DB_train_*, z)    *ψ_cur_* ← select(*Ψ_s_*, *|ψ.*latent|= z)    **if** *ψ_cur_ =* ∅ **then**
      *Ψ_s_*.add(*ψ_new_*)    **else**
      *Y* ← *ψ_new_*.encoder.propagate(*DB_test_*)       *X’* ← *ψ_new_*.decoder.propagate(*Y*)       *r_new_ ←* computeReconstructionError (*DB_test_*, *X’*)       *Y* ← *ψ_cur_*.encoder.propagate(*DB_test_*)       *X’* ← *ψ_cur_*.decoder.propagate(*Y*)       *r_cur_ ←* computeReconstructionError (*DB_test_*, *X’*)       **if** avg(*r_new_*) *<* avg(*r_cur_*) **and** max(*r_new_*) *<* max(*r_cur_*) **then**
         *Ψ_s_*.replace(*ψ_cur_, ψ_new_*)  **return**
*Ψ_s_*

The procedure starts by splitting the data in DB in both training and testing datasets, e.g., following a typical 80–20% split [32] (line 1 in Algorithm 2). Then, each LS size *z* in *Z* is selected and a new AE *ψ_new_* is trained for such LS size (lines 2–3). This new AE needs to be compared against the current one in the pool with the same LS size (denoted as *ψ_cur_*) and therefore, it is retrieved from the pool (line 4). Note that *ψ_new_* is directly added to the pool if there is no currently available AE with such size *z* (lines 5–6). Otherwise, the testing dataset is used to evaluate the reconstruction error in both *ψ_new_* and *ψ_cur_* (lines 8–13). Thus, the current AE is replaced by the new one if both average and maximum reconstruction errors are reduced by the new AE (lines 14–15). Eventually, the updated AE pool is returned.

### 4.3. SS-AD

Algorithm 3 details the pseudo-code of the SS-AD procedure that runs locally in the DA every time a new compressed telemetry stream is obtained and hence, a new reconstruction error vector *r_st_* is available. Since the principle of anomaly detection using AEs relies on the fact that an anomalous input will be poorly reconstructed, an anomaly error detection threshold *ε_anom_* is needed to perform such detection. Indeed, anomaly detection is triggered if either one of the following conditions is met: (*i*) a number α of consecutive measurements produced a reconstruction error larger than threshold *ε_anom_* or (*ii*) a number *β* of total measurements (non-consecutive) produced a reconstruction error larger than threshold *ε_anom_*.

The algorithm starts by initializing the counters of the consecutive and total number of measurements above the error threshold (line 1 in Algorithm 3). Then, each single error value in the *r_st_* vector is evaluated and compared with the threshold (lines 2–3). When the error exceeds the thresholds, then both counters are increased in one unit (lines 4–5). At this point, it is worth checking if one of the anomaly detection conditions is met and if so, the procedure stops and returns an anomaly detection event (lines 6–7). Therefore, it is necessary to reset the counter of consecutive values above the threshold before analyzing the next error value (lines 8–9). Finally, no anomaly event is returned in the case that none of the conditions is met (line 10).
**Algorithm 3.** SS-AD**INPUT**: *r_st_*, *ε_anom_, α, β* **OUTPUT**: *anomaly*1. 2. 3. 4. 5. 6. 7. 8. 9. 10.*k_cons_, k_total_* ← 0    **for each** *r* ∈ *r_st_* **do***:*
   **if** *r > ε_anom_*
**then**
      *k_cons_* ← *k_cons_* +1       *k_total_* ← *k_total_* +1       **if** *k_cons_ == α*
**or** *k_total_ = β*
**then**
         **return** *True*
   **else**
      *k_cons_* ← 0  **return** *False*

### 4.4. MS-AGD

The pseudo-code of the MS-AGD procedure is detailed in Algorithm 4, which aims at computing a score that increases when a number of sensors within a group generate a high reconstruction error at the same time. Indeed, this score has the form of a vector of w positions, indicating the score at a given time unit within the analyzed monitoring interval (which allows fine multiple anomaly detection analysis). Moreover, recall that the MS-AGD can be executed at the device level, e.g., analyzing all (or a subset) of the sensors of a given device, or at the cluster level, e.g., analyzing all (or a subset) of the sensors in a given cluster. Regardless of the case, let us consider that the reconstruction error vectors obtained at a given monitoring time interval of a given group of sensors are denoted as R. This is the main input of MS-AGD, which also requires the specific parameter γ that defines the time interval size needed to compute the score.

The first step is to initialize the score vector, as well as the auxiliary matrix Q that is going to facilitate score computation (lines 1–2 of Algorithm 4). In particular, Q is a sparse 0–1 matrix, where cell <*i*, *j*> is 1 if and only if the sensor *i* at time unit *j* took a measurement above the average value of that sensor within monitoring interval *t*. After computing Q (lines 3–9), the score is computed for every time unit within the monitoring interval (lines 10–13). The score of each time unit *i* is the product of components a and b. On the one hand, a is the normalized sum of 1 s in Q that time, i.e., which proportion of sensors generates a measurement that produced a reconstruction error above the average. On the other hand, b computes the normalized dot product of Q in the last γ time units. Note that a large value indicates that there are consecutive time units where several sensors are above the average. In particular, b = 1 when all sensors in S_g_ stay above average reconstruction error during a consecutive number γ of time units.
**Algorithm 4.** MS-AGD**INPUT**: *R* = {*r_st_*, ∀*s* ∈ *S_g_*}, γ **OUTPUT**: *score*1. 2. 3. 4. 5. 6. 7. 8. 9. 10. 11. 12. 13. 14.*score* ← zeros(*w*) *Q* ← zeros(|*S_g_*|, *w*) *i* ← 0 **for** s ∈*S_g_* **do**
   *i* ← *i* + 1    *r_avg_* ← avg(*R*.*r_st_*)    **for** *j == 1*..w **do**
      **if**
*R*.*r_st_*[*j*] > *r_avg_* **then**
         *Q*[*i*,*j*] ← 1  ** for** *j ==* γ..*w*
**do**
   *a* ← sum(*Q*[:, *j*])/|*S_g_*|    *b* ← dotproduct(*Q*[:,*j*-γ+1:*j*])/γ    *score*[*j*] ← *a·b*
 **return**
*score*

**Figure 3:**
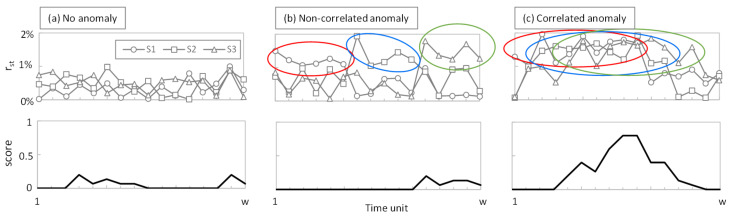
Example of the MS-AGD score: no (**a**), non-correlated (**b**), and correlated (**c**) anomaly.

To better understand the rationale behind the MS-AGD score, Figure 3 shows the reconstruction error r_st_ and the score in a monitoring time interval of w = 20 of an example with three sensors. Three different cases are depicted, assuming γ = 5: (i) the error stays constant and low for all the time and sensors (no anomaly, Figure 3a); (ii) the error increases in all the sensors but not at the same time (non-correlated subtle anomaly, Figure 3b); and (iii) the error increases in all the sensors and partially coincides in time (correlated subtle anomaly, Figure 3c). For the sake of simplicity, the average reconstruction error is around 0.5% in all the sensors in Figure 3a and around 1.5% in all the sensors in Figure 3b,c. Colored circles indicate when the reconstruction error is above the threshold. As can be observed, the score reaches significant values (above 0.5) only when several sensors exceed the average reconstruction error at the same time.

## 5. Performance Evaluation

In this section, we first introduce the simulation environment developed to evaluate the methods and algorithms presented in previous sections. Then, we analyze the performance of AAC, SS-AD, and MS-AGD using telemetry data from a real physical system. Finally, we analyze the impact of the proposed methods on a network case study where transport network capacity savings are shown.

### 5.1. Simulation Environment

**Figure 4:**
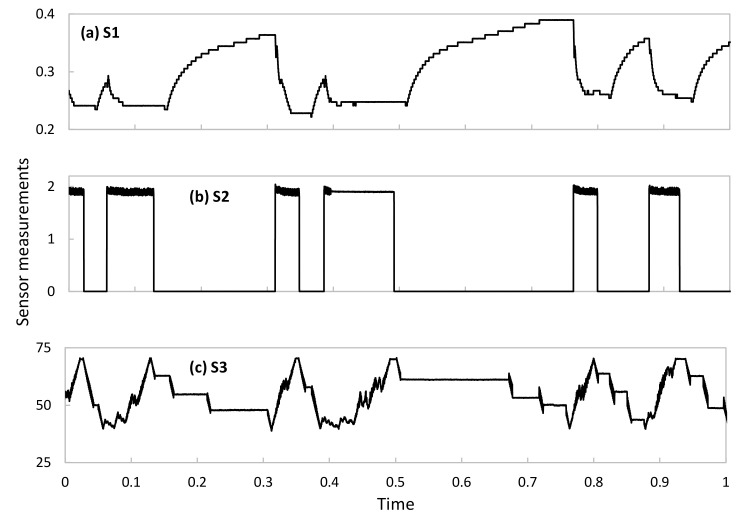
Example of the sensor data time series processed by each DA.

For numerical evaluation purposes, we implemented a Python-based simulator reproducing the main blocks of the architecture presented in Figure 2, as well as the algorithms in Section 4. In particular, a CA with three Das was configured, where every DA processes data from one single sensor. Sensors were implemented as time series data generators injecting real measurements (one per second) from the Water Distribution (WADI) dataset [33]. The WADI dataset contains experimental sensor data measured in a water distribution testbed under different conditions, including normal operation and operation in the presence of system perturbations. The testbed comprises several water tanks as well as chemical dosing systems, booster pumps, valves, instrumentation, and analyzers, thus forming a complete and appropriate physical system for the performance evaluation of the proposed methods. Among all available data in WADI, we selected three time series from three different sensor types (hereafter, referred to as *S1*, *S2*, and *S3*) with different behaviors and patterns. Specifically, the selected sensors are located in a water pressure valve and collect measurements of pressure, volume, and voltage. Figure 4 shows an example of each sensor time series data under normal operation. As can be observed, they are different in terms of time patterns, as well as in the magnitude and range of the telemetry data. Note that these data cover typical and widespread patterns observed in telemetry data, which will allow extending this performance evaluation analysis to other DT-based systems such as smart manufacturing and communication networks.

For the sake of simplicity, we assume that AEs in the pool of CA and Das are trained using Algorithm 2 after a period of raw data collection to populate the initial database *DB*. Without loss of generality, we assume that the measurements collected during this period belong to the normal operation of the physical system. Then, fixing interval *w* to 256 s, we obtained 7.68e5 samples for training, as well as 9.6e4 samples for testing. Regarding AE pool configuration, we considered four different AEs with *Z =* {4, 8, 16, 32} LS sizes. In all the cases, we considered two hidden layers, with 128 and 64 hidden neurons each. We used the *keras* library for AE training and testing, as well as *pandas* and *numpy* to load and manipulate the datasets. AEs were trained during 100 epochs using the *adam* optimizer and mean absolute errors as loss function, which results in reconstruction accuracy values around 99%.

### 5.2. AAC Performance

**Figure 5:**
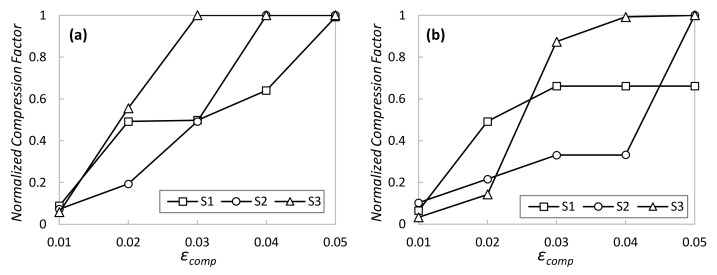
AAC performance under normal operation (**a**) and operation with perturbations (**b**).

The first numerical study is focused on evaluating the performance of the AAC procedure in Algorithm 1 once the AE pool of every DA has been trained with the telemetry data of its specific sensor. Figure 5 shows the compression factor as a function of target reconstruction error *ε_comp_* for both normal operation (Figure 5a) and operation with perturbations (Figure 5b) after 9 h of simulated time (~32,000 monitoring samples per sensor). The compression factor was normalized between 0 and 1, where 0 means that the AAC cannot compress any measurement below the target *ε_comp_* and 1 means that all measurements are compressed with the AE with the lowest LS size (in our case, 4). As can be observed, the AAC shows the desired adaptability, sharply increasing the compression factor when *ε_comp_* is relaxed. Interestingly, we can observe that different time series produce different compression performances, even when AEs were specifically trained for that data. However, maximum compression is always achieved with low reconstruction error (0.05) under normal system operation and remains very high when perturbations appear in the system, which validates the applicability of the proposed method in systems subject to changes in the telemetry data generated.

**Figure 6:**
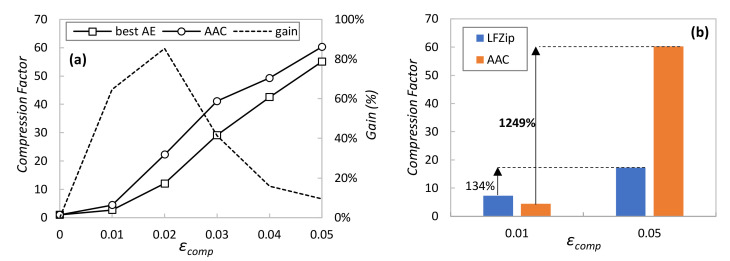
AAC benchmarking against the best single AE (**a**) and LFZIP (**b**) methods.

Once the AAC has been presented as an adaptive and polyvalent method, let us now focus on evaluating its performance compared to the two benchmarking methods. Firstly, Figure 6a compares AAC against the simplest method consisting of the single AE that works better for a given *ε_comp_*, i.e., the one with the smallest LS size that always achieves a reconstruction error less than *ε_comp_*. Note that this benchmarking method is easy to deploy in our system, provided that the required *ε_comp_* does not (often) change in time, because every requirement variation could entail a new AE re-training to adjust LS size. The figure shows the absolute compression factor (not normalized), as well as the relative gain of AAC with respect to using the best AE in each case. In light of the results, we can conclude that AAC produces a larger compression ratio than using a single AE, reaching a remarkable relative gain above 60% for stringent reconstruction errors around 0.01. Recall that AAC can adapt to changes in *ε_comp_* without the need of retraining AEs; that, combined with its high performance, makes AAC the best option for AE-based compression.

In order to have a second benchmarking evaluation, Figure 6b compares the achieved compression ratio for two selected *ε_comp_* values against the compression method presented in [14], called LFZip. Similar to AAC, LFZip is a lossy compression method using fully connected neural network decoders that achieves good compression ratios. In [14], the authors provide the achieved compression ratio for the selected target reconstruction errors using different time series data. The figure compares the performance of AAC (averaging all sensors under normal operation) and LFZip (results from [14]), where the large benefits of AAC can be observed. However, since we were not able to reproduce either the LFZip method with our sensor data or AAC with the data in [14] (due to the lack of algorithm details and data availability), the conclusions of such comparison are mild. For this reason, we included the relative gain of each method when *ε_comp_* is relaxed from 0.01 to 0.05. In view of the values, we can state that AAC clearly outperforms LFZip in terms of adaptability to variable requirements and relative compression gain.

**Figure 7:**
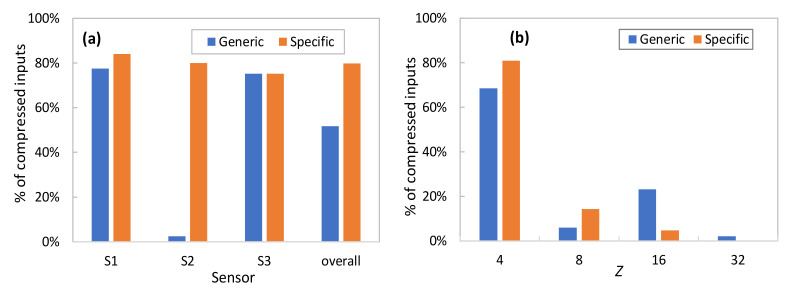
Generic and specific AAC performance vs. sensor type (**a**) and latent space size (**b**).

It is worth noting that the outstanding AAC performance illustrated so far requires the availability of a pool of aEs specifically trained for each of the sensors. Once a new sensor is installed in the physical system and telemetry is starting to be collected and processed by a new or existing DA, such specific aEs are not available until enough data have been collected. This is the reason why, as introduced in previous sections, our approach proposes initializing the AAC with a pool of generic aEs trained with heterogeneous data, i.e., a mix of data from other sensors available in the data lake. Figure 7a compares the percentage of compressed samples using generic aEs trained with a mix of telemetry measurements of all sensors and specific aEs for each of the sensors individually. In both cases, the AAC has been configured with a stringent *ε_comp_* = 0.01. As can be seen, generic aEs produce an overall good performance (around 50% of samples can be effectively compressed), although this provides a negligible benefit for sensors that behave very differently from the considered generic data. This occurs in S2 data, showing a clear on-off period (recall the example in Figure 4) that vastly differs from the generic data used for training. As soon as specific AEs can be trained, then both individual and overall compression increases (around 80% of samples can be compressed).

Finally, Figure 7b details how many times every AE in the pool is used, for both generic and specific AE pools. Results show the average performance for all DAs and *ε_comp_* = 0.01. Note that the smallest LS size is frequently selected; however, sometimes a smaller compression (larger LS) is needed to guarantee the target reconstruction error, which adds value to the proposed AAC method. Moreover, the use of larger AEs is reduced when specific AEs are trained. For this very reason, we can conclude that the use of generic AEs is useful to provide compression from the beginning of sensor operation but needs to be substituted by specific AEs to reach maximum performance.

### 5.3. SS-AD and MS-AGD Performance

Once the AAC has been numerically evaluated and validated, in this section, we focus on evaluating the performance of anomaly detection procedures assuming that specific AE are already trained and working. In particular, we configured our simulator to reproduce two different use cases: (*i*) large individual anomalies for SS-AD evaluation and (*ii*) subtle time-correlated anomalies for MS-AGD evaluation.

For the first use case, we assume that SS-AD is continuously running for each sensor during 9 h of normal operation followed by a drastic change in the pattern of the generated data (happening at time *t_anom_*). In order to introduce a variety of anomalies, we consider that sensor *Si* starts generating at *t_anom_* data similar to that of sensor *Sj*, being *i*≠*j* and *i*, *j* ∈ {1, 2, 3}, thus reproducing six different anomalies.

**Figure 8:**
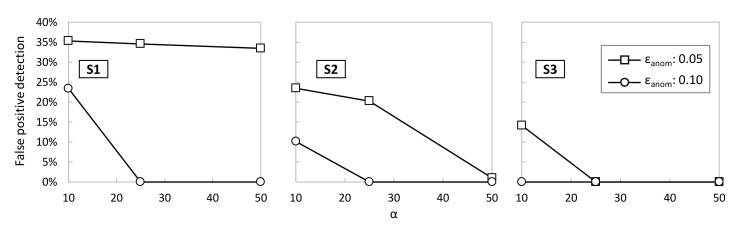
SS-AD: false positive detection.

Figure 8 evaluates the percentage of false positives detected by each of the sensors as a function of different values of SS-AD parameters *ε_anom_* and α (β was fixed to 100). A false positive is detected if SS-AD returns True during the period of normal operation, i.e., when no anomalies are introduced. In light of the results, we can conclude that increasing *ε_anom_* to 0.10 is sufficient to reduce α to short values (25 for *S1* and *S2* and 10 for S3) that lead to zero false positive detections. Note that, the shorter α is, the faster the detection of true anomalies. Then, assuming the best configuration of parameters for every sensor, Table 3 shows the detection accuracy of all the aforementioned anomalies. It is worth noting that SS-AD achieves very high accuracy (>95%) for most of the considered anomalies. Indeed, only the *S1* SS-AD process is not able to detect *S3*-like data, which is reasonable due to the similarity of both the *S1* and *S3* time series. Therefore, we can conclude that SS-AD performs accurate and robust detection of individual anomalies.

**Figure 9:**
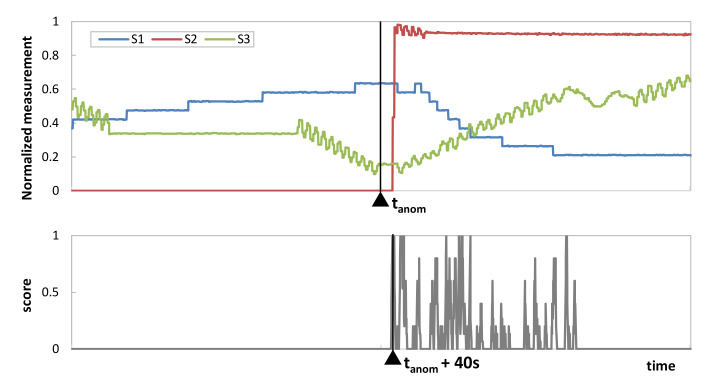
MS-AGD performance.

Regarding the second use case, we took advantage of WADI dataset measurements collected under perturbations that were intentionally introduced in the system. The available metadata clearly indicates the time when a perturbation starts, which we identified as *t_anom_*. Figure 9 plots the three sensors’ data in the period before and after *t_anom_*, as well as the score computed in all such periods. In view of the results, we can conclude that the proposed score clearly identifies when the correlated anomaly starts (no false positive detection is observed before *t_anom_*). Note that the first time interval where the score reaches a value significantly larger than 0 is only 40 s later than *t_anom_*, which validates MS-AGD as a prompt time-correlated anomaly detection method.

### 5.4. Case Study

Eventually, we conducted a numerical case study in order to evaluate the impact of the proposed methodology assuming a larger network scenario such as the one sketched in Figure 1a. Thus, we assume that a physical system containing hundreds to thousands of sensors is geographically distributed among a number of locations where the DAs are locally deployed. For the sake of simplicity, let us assume that the overall telemetry data generated by all the sensors in the system, i.e., the total volume that needs to be gathered by the DT, is fixed at 400 Gb/s. Moreover, let us assume an optical transport network that allows transparent connectivity between the remote physical locations and the location where the DT is deployed, e.g., a data center. To support the transport of such telemetry data, optical connections taking advantage of digital subcarrier multiplexing technology can be deployed [34]. This ensures that optical connections can be established with a fine granularity of 25 Gb/s each.

**Figure 10:**
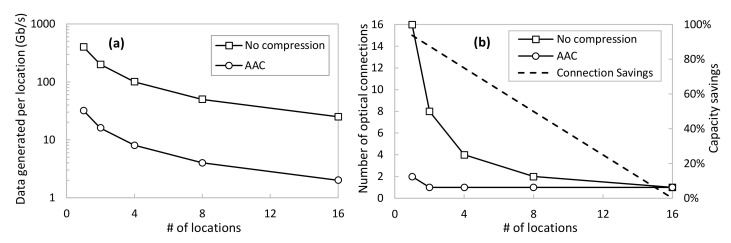
Network study analysis: data generated (**a**) and number of optical connections (**b**).

Figure 10a shows the amount of data injected as a function of the number of locations, assuming an even split among locations of the total amount of telemetry data. Two cases are shown: no compression and using AAC. For the latter, we consider *ε_comp_* = 0.01 and, according to Figure 6 and considering that the sensors behave similarly to the ones used before, the average compression factor is around 12.5. Assuming this compression performance, the figure shows great savings in the total amount of data generated by every location distributed in the network. Nevertheless, the impact on the true amount of data that needs to be conveyed in the transport network will depend on the number of optical connections needed to carry out such data. This is shown in Figure 10b as a function of a number of locations, as well as the capacity savings of using AAC with respect to the no compression scenario. For instance, 50% of optical capacity savings are achieved when 400 Gb/s of raw data are generated among eight different locations. In this case, every location is generating around 50 Gb/s of raw telemetry data, which requires two optical connections between the location and the centralized DT. On the contrary, the proposed AAC method reduces the conveyed data to 4 Gb/s, which can be served with only one optical connection per location.

Hence, we can definitively conclude that the proposed adaptive telemetry compression mechanism allows a large reduction in the number of optical connections and true data to be conveyed through the transport network.

## 6. Conclusions

In this paper, we presented a smart management system of DT telemetry data consisting of different processes for adaptive telemetry data compression (AAC), single sensor anomaly detection (SS-AD), and multiple sensor anomalous group diagnosis (MS-AGD). All the methods made use of AEs trained with generic and specific sensor telemetry data, as well as a set of algorithms that used those AEs to maximize the performance of compression and anomaly detection.

The numerical evaluation of such models and algorithms was performed using an experimental data set from a water distribution system. The main conclusions derived from such numerical analysis are (*i*) AAC produces a larger compression ratio than using a single AE, reaching a remarkable relative gain above 60% for stringent reconstruction errors around 1%; (*ii*) AAC achieves compression ratios one order of magnitude larger than other benchmarking lossy compression mechanisms in the literature; (*iii*) SS-AD achieves an anomaly detection accuracy larger than 95% when telemetry data anomalies are injected; and *iv*) MS-AGD is able to accomplish the prompt detection (<1 min) of subtle correlated anomalies affecting a group of sensors.

In addition, the proposed smart management of telemetry data for the DT use case was evaluated in terms of the reduction in transport network resources. To this aim, we considered distributed scenarios where telemetry data sources were spread among different network locations, thus needing to gather such telemetry data in a centralized location. Results showed that remarkable capacity savings, measured in terms of dedicated optical connections, were achieved for moderately-high distributed scenarios.

As a final remark, it is worth mentioning that this work allows for promoting the deployment of DT-based management solutions for those industrial systems that have not yet adopted it. Since telemetry data sources are currently available (sensors in automated control systems are widely used in industry), one of the major current obstacles for migrating towards DT-based solutions is the high cost of the management and curation of such a large amount of generated telemetry data. In this regard, the proposed contributions showed a significant reduction in such cost by efficient compression and decentralized analysis, thus facilitating the adoption of DT in industry.

## Figures and Tables

**Table 2:** Notations.

*S*	Set of sensors
*G*	Set of groups. A group comprises a set of sensors that can be the sensors in a given device or the sensors of the same type in a given cluster
*S_g_⊂ S*	Subset of sensors belonging to group g⊂ G
*Z*	Set of allowable sizes for the LS
*w*	Monitoring interval duration, in time units
*Ψ_s_*	Pool of AEs for compressing telemetry data from sensor s
*x_st_*	Raw telemetry data vector from sensor s at time interval t
*y_st_*	Compressed telemetry data vector from sensor s at time interval t
*x’_st_*	Reconstructed telemetry data vector from sensor s at time interval t
*id_st_*	Id of the AE used to compress data from sensor s at time interval t
*r_st_*	Reconstruction error vector from sensor s at time interval t
*DB*	Telemetry Database for training and testing purposes
*ε_comp_*	Target average reconstruction error for compression
*ε_anom_*	Individual reconstruction error for anomaly detection
*α*	Number of consecutive error values above anomaly detection threshold
*β*	Number of total error values above anomaly detection threshold

**Table 3:** SS-AD: best configuration and anomaly detection accuracy.

Si	ε_anom_	α	Sj		
			S1	S2	S3
S1	0.1	25	-	95.7%	0%
S2	0.1	25	95.4%	-	99.9%
S3	0.05	25	95.6%	95.5%	-

## Data Availability

Not applicable.
